# Mutant p53 tunes the NRF2-dependent antioxidant response to support survival of cancer cells

**DOI:** 10.18632/oncotarget.24974

**Published:** 2018-04-17

**Authors:** Kamil Lisek, Elena Campaner, Yari Ciani, Dawid Walerych, Giannino Del Sal

**Affiliations:** ^1^ National Laboratory CIB, Area Science Park Padriciano, Trieste 34149, Italy; ^2^ Department of Life Sciences, University of Trieste, Trieste 34127, Italy; ^3^ Mossakowski Medical Research Centre, Polish Academy of Sciences, Warsaw 02-106, Poland; ^4^ Present address: Max-Delbrück-Centrum for Molecular Medicine, Berlin 13092, Germany

**Keywords:** NRF2, mutant p53, cancer, oxidative stress

## Abstract

NRF2 (NFE2L2) is one of the main regulators of the antioxidant response of the cell. Here we show that in cancer cells NRF2 targets are selectively upregulated or repressed through a mutant p53-dependent mechanism. Mechanistically, mutant p53 interacts with NRF2, increases its nuclear presence and resides with NRF2 on selected ARE containing gene promoters activating the transcription of a specific set of genes while leading to the transcriptional repression of others. We show that thioredoxin (*TXN)* is a mutant p53-activated NRF2 target with pro-survival and pro-migratory functions in breast cancer cells under oxidative stress, while heme oxygenase 1 (*HMOX1)* is a mutant p53-repressed target displaying opposite effects. A gene signature of NRF2 targets activated by mutant p53 shows a significant association with bad overall prognosis and with mutant p53 status in breast cancer patients. Concomitant inhibition of thioredoxin system with Auranofin and of mutant p53 with APR-246 synergizes in killing cancer cells expressing p53 gain-of-function mutants.

## INTRODUCTION

The transcription factor nuclear factor erythroid 2-related factor 2 (NRF2) is the main and evolutionary conserved regulator of the antioxidant pathways in the cell. In response to oxidative stress, the inhibitor Keap1 cannot degrade NRF2, and newly synthesized NRF2 accumulates, translocates to the nucleus and drives the transcription of ARE containing genes [1]. The role of the NRF2-Keap1 pathway in cancer initiation, progression and chemoresistance has been studied extensively in the recent years. Keap1 mutations activating NRF2 or mutations in NRF2 gene itself are frequent events in many cancer types [2, 3]. Moreover, various oncogenes have been reported to impact the NRF2 pathway by upregulating total mRNA and protein levels of NRF2 [4, 5]. NRF2 controls key components of endogenous antioxidant systems in both cancer and normal cells [1, 6]. Until now over 500 genes have been reported to be under NRF2 control [7]. While in normal cells the NRF2/Keap1 antioxidant pathway plays a crucial role in maintaining cellular homeostasis and preventing tumorigenesis [1], in cancer cells high expression of NRF2-regulated genes provides them with cytoprotection, contributing to their oncogenic capabilities [8, 9].

Mounting evidence links the oncogenic activities of mutant p53 [10] to different aspects of NRF2 transcriptional activity [11] although reported results open a number of questions on the interplay between these two factors, as mutant p53 was shown to repress the elements of the NRF2-dependent oxidative stress response [12], while other researchers showed that mutant p53 activates *NRF2* gene transcription [13]. We have previously demonstrated that while in cancer cells mutant p53 activates proteasome genes in cooperation with NRF2, it indeed simultaneously represses another NRF2 target gene - heme oxygenase 1 (*HMOX1*) [14]. The physiological significance of this mutant p53-dependent bi-directional NRF2 target regulation in cancer cells has remained unclear.

Here, we investigate how mutant p53 impacts on NRF2 activity acting as a molecular switch that turns on- or off- specific components of the NRF2 transcriptional program thus tuning NRF2 activity in cancer cells. We also provide evidence that simultaneous targeting of mutant p53 and of the thioredoxin system by combining APR-246/PRIMA-1MET and Auranofin, synergizes providing a therapeutic advantage against breast cancer cells.

## RESULTS

### Mutant p53 differentially regulates NRF2 transcriptional targets

In order to evaluate the impact of mutant p53 on the expression of NRF2 transcriptional program, we perused already available datasets to sort out a signature consisting of well established NRF2 target genes which represent various biological processes important for normal and cancer cell metabolism. We selected key NRF2 downstream targets regulating the antioxidant systems of the cell, such as glutathione (*GCLC, GCLM*), thioredoxin (*TXN, TXNRD1*), as well as genes encoding for: cystine antiporter (*SLC7A11*), quinone oxidoreductase (*NQO1*), heme oxygenase 1 (*HMOX1*), multidrug resistance proteins (*ABCC3, ABCC5*), proteasome subunits (*PSMA2, PSMC1, PSMD10*) and phosphoserine aminotransferase (*PSAT1*) [7, 15–17].

**Figure 1:**
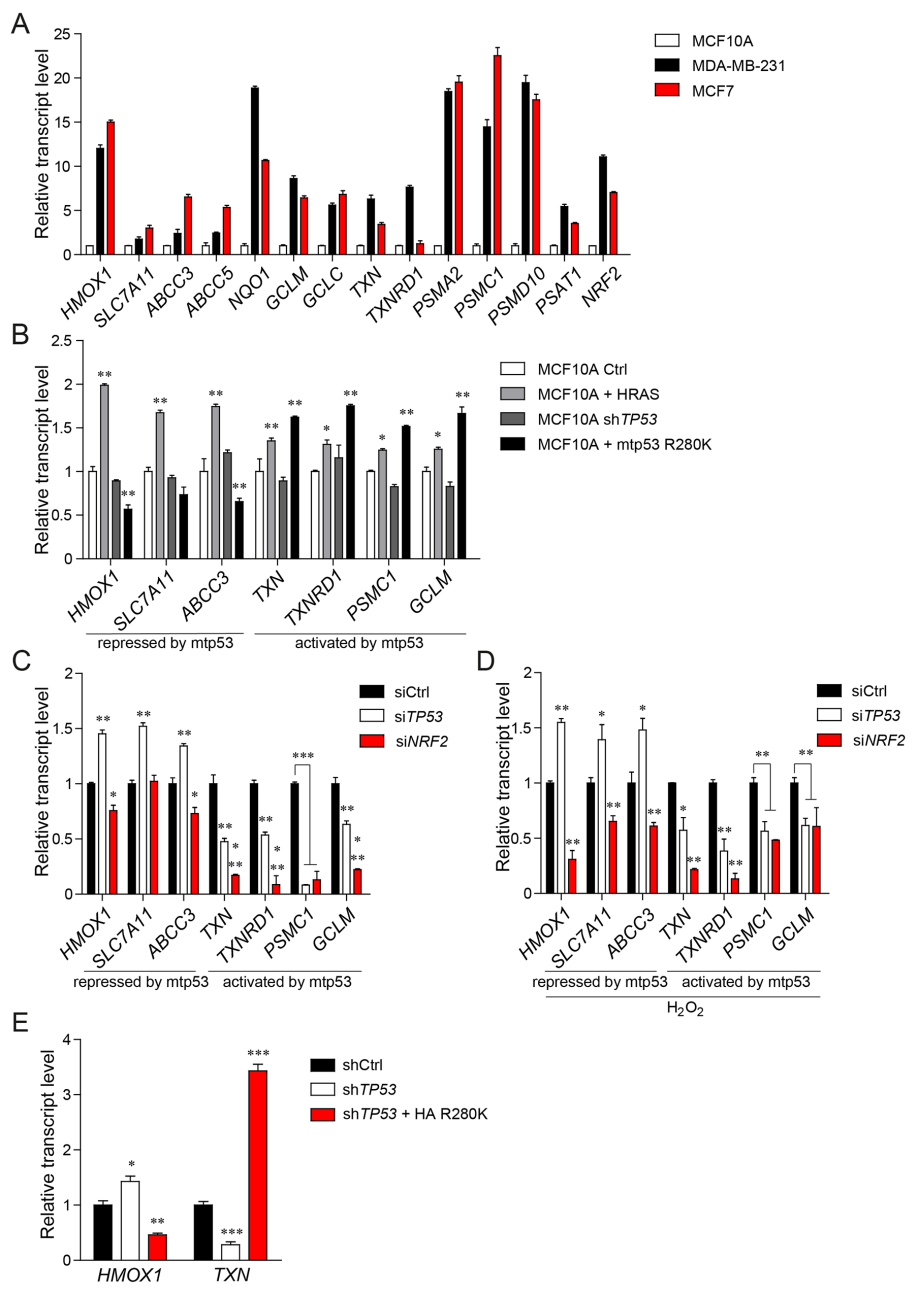
Mutant p53 differentially regulates the expression of NRF2 transcriptional targets **(A)** Expression of selected NRF2 targets in breast cancer cell line MDA-MB-231 (bearing mutant p53 R280K), breast cancer cell line MCF7 (bearing wt p53) in comparison with normal epithelial breast cell line MCF10A (bearing wt p53). **(B)** Expression of NRF2 targets in MCF10A cells. Normal epithelial cells (MCF10A) stably transfected with empty retroviral vector (Ctrl), vector encoding oncogenic variant H-Ras G12V (HRAS), vector encoding shRNA targeting *TP53* transcript (sh*TP53*), and mutant p53 CDS shRNA-resistant HA-tagged variant R280K, stably introduced into the MCF10A sh*TP53* cell line (+mtp53 R280K). **(C,D)** Effect of mutant *TP53* or *NRF2* silencing on the selected NRF2 targets in MDA-MB-231 cells in unstressed conditions (C) or under oxidative stress induced by incubation of MDA-MB-231 cells with 500μM H_2_O_2_ for 12 hours (D). **(E)** Expression levels of *HMOX1* and *TXN* in MDA-MB-231 cells stably silenced for control (shCtrl), *TP53* (sh*TP53*), or *TP53* together with stable mutant p53 R280K overexpression (sh*TP53* + HA R280K). Data shown in A-E are the means ± s.d. of n=3 independent experiments, ANOVA test with Bonferroni correction: ^*^ p<0.05, ^**^ p<0.01, ^***^ p<0.001. Expression levels are given relative to *ACTB*.

In order to understand to what extent the NRF2 transcriptional profile differs between normal and cancer cells of the breast epithelium, we compared MDA-MB-231 (a breast cancer cell line bearing missense mutant p53), MCF7 (a breast cancer cell line harboring wild-type p53) and MCF10A (untransformed, immortalized breast epithelial cells) for basal expression of the NRF2 gene signature (Figure 1A). Breast cancer cells (MDA-MB-231 and MCF7) showed a markedly higher expression of NRF2 and of all investigated NRF2 targets than normal cells (MCF10A), confirming previous observations from other cellular models [5, 18, 19].

We expected from our previous study that mutant p53 may differentially affect subsets of NRF2 targets [14]. Hence, we compared the expression of selected NRF2 target genes between normal breast epithelial MCF10A cells bearing wild-type p53 (wtp53), MCF10A cells silenced for *TP53* (MCF10A^+sh*TP53*^) and MCF10A cells silenced for wild-type *TP53*, ectopically overexpressing the oncogenic mutant p53 (mtp53) R280K (MCF10A^+mtp53 R280K^). In this experimental setup we observed that in the presence of mutant p53 only a subset of the NRF2 transcriptional program is upregulated, while other NRF2 targets are repressed. MCF10A^+mtp53 R280K^ expressed higher levels of a proteasome subunit gene (*PSMC1*), of thioredoxin system genes (*TXN*, *TXNRD1*), and of glutathione (*GCLM*) in comparison to normal MCF10A cells, while the expression of targets such as *HMOX1*, *SLC7A11*, *ABCC3* was repressed in the presence of the mutant p53 gain of function (GOF) variant (Figure 1B).

Interestingly, in MCF10A cells stably expressing the oncogenic variant of HRAS (MCF10A^+HRAS^), expression of all investigated NRF2 targets was significantly upregulated, confirming previous reports in different cellular systems indicating that other oncogenes (i.e. oncogenic alleles of *KRAS, HRAS, BRAF* or *CMYC*) have an overall activating effect on the NRF2 transcriptional activity [5] (Figure 1B).

We confirmed the differential regulation of NRF2 transcriptional targets by mutant p53 also in MDA-MB-231 breast cancer cells in which we silenced mutant *TP53* expression with siRNAs [14, 20]. In this context, we observed a downregulation of *PSMC1*, *TXN*, *TXNRD1*, and *GCLM*, and an upregulation of *HMOX1*, *SLC7A11* and *ABCC3* transcription (Figure 1C and Supplementary Figure 1A). Similarly, in p53-null H1299 lung cancer cells upon stable introduction of mutant p53 variant R175H, we observed an upregulation of *TXN* and *TXNRD1* and a concomitant downregulation of *HMOX1* and *ABCC3* transcription (Supplementary Figure 1B). Silencing of *NRF2* abolished mutant p53 ability to sustain *TXN* and *TXNRD1* mRNA levels indicating that mutant p53 requires NRF2 to promote the transcription of these genes.

Since NRF2 plays a major role as an antioxidant response regulator in cancer and in normal cells we sought to test if the regulation of the expression of NRF2 targets by mutant p53 is maintained also under the oxidative stress conditions. We first tested different oxidative stress inducers in promoting NRF2 activation in MDA-MB-231 cells by analyzing both NRF2 localization and the expression levels of its targets. As shown in Supplementary Figure 2A, 2B, H_2_O_2_, menadione and sodium arsenite induced NRF2 nuclear localization and transcription of its targets in a similar manner. Considering the reported toxic effect of menadione and sodium arsenite [21–24], we selected H_2_O_2_ as oxidative stress inducer for the next experiments. We then treated MCF10A^+ RAS^, MCF10A^+mtp53 R280K^ or MDA-MB-231 cells with H_2_O_2_ and observed that NRF2 targets and NRF2 itself are transcriptionally activated in response to exogenous oxidative stress stimuli and that mutant p53 regulates NRF2 targets in a similar way as in unstressed conditions (Figure 1D and Supplementary Figure 2C, 2D). Abrogating the expression of *NRF2* with siRNA inhibits NRF2 target expression in both normal and oxidative stress conditions (Figure 1C, 1D and Supplementary Figure 2C, 2D).

Next, we validated the effect of mutant p53 on NRF2 transcriptional program performing rescue experiments in MDA-MB-231 cells in which we stably silenced mutant *TP53* (MDA-MB-231^+sh*TP53*^). We investigated *TXN* as a representative of NRF2 targets activated by mutant p53, and *HMOX1* among NRF2 targets repressed by mutant p53. In this setup, we observed downregulation (at both mRNA and protein levels) of *TXN*, and upregulation of the mRNA level of *HMOX1* (Figure 1E and Supplementary Figure 2E). Reintroducing HA-tagged mutant p53 in these cells (MDA-MB-231^+shp53/+HA R280K^) we observed a rescue of *TXN* gene expression and of the corresponding thioredoxin (Trx) protein level, coupled with the transcriptional repression of *HMOX1* (Figure 1E and Supplementary Figure 2E). Heme oxygenase 1 (HO-1) protein levels were undetectable by western blot analysis in unstressed conditions (data not shown).

We next evaluated whether the effect of mutant p53 on NRF2 targets involved a modulation of NRF2 protein levels. We performed both ectopic expression of two *TP53* mutants in MCF10A and silencing of mutant *TP53* in different cell lines (MDA-MB-231, MDA-MB-468, BT549 and SUM149) and analyzed NRF2 levels. As shown in Supplementary Figure 3A-3C modulation of mutant p53 did not have a strong impact on NRF2 protein levels. Taken together these results indicate that mutant p53 exerts both positive and negative control over NRF2 transcriptional activity without altering the NRF2 protein levels.

### Mutant p53-NRF2 interaction is crucial for regulating NRF2 targets in mutant p53 bearing cancer cells

**Figure 2:**
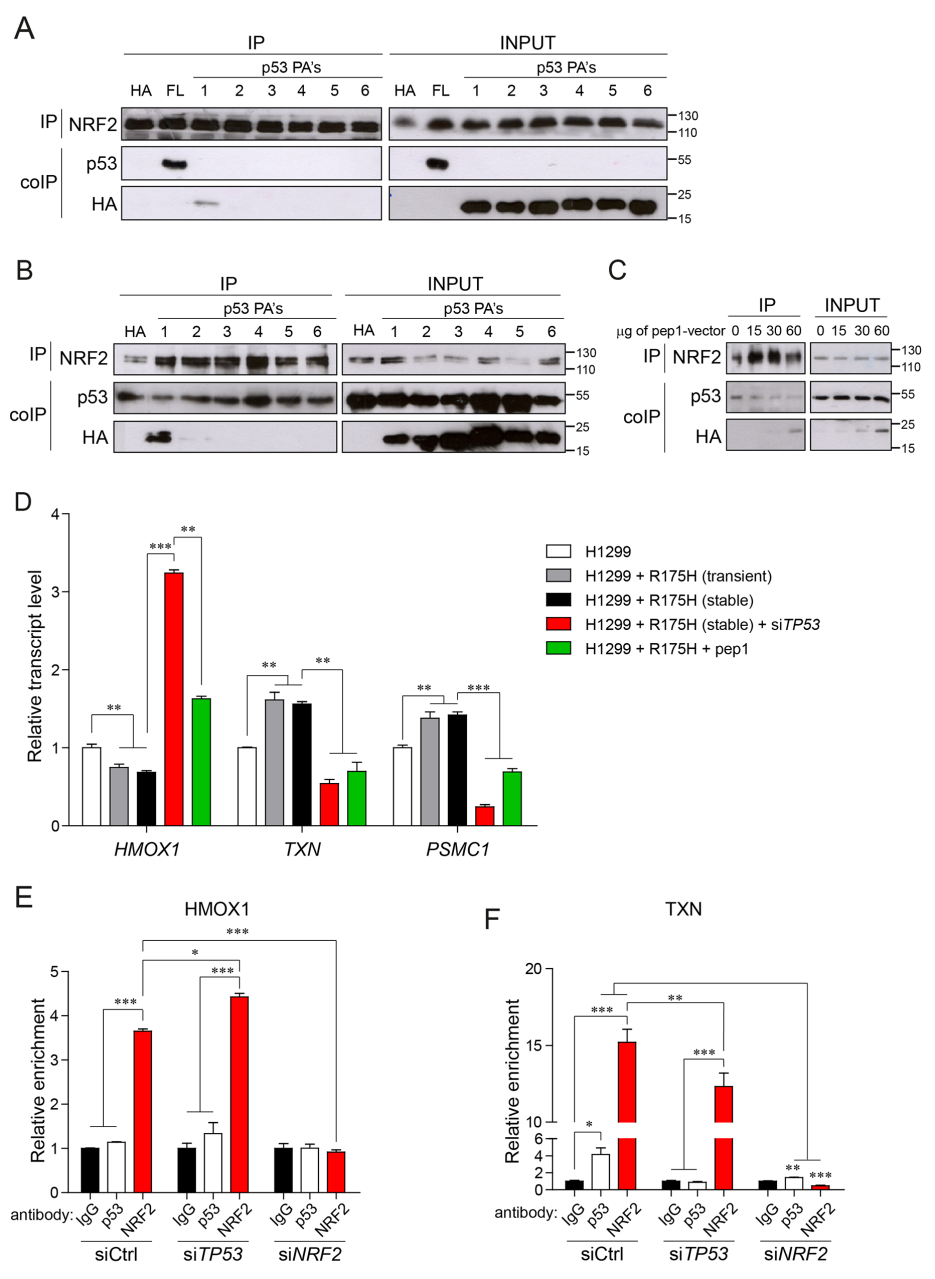
Mutant p53-NRF2 interaction is crucial for regulating NRF2 targets **(A)** Western blot analysis of co-immunoprecipitation (coIP) of NRF2 with p53R175H and with p53 peptide aptamers (p53 PA’s) in lysates from H1299 cells upon transient transfection with empty- (HA), p53R175H- (FL) or p53 PA vectors. NRF2-antibody immunoprecipitated proteins (IP) and input cell lysates (INPUT) were analyzed by western blot with the indicated antibodies; size markers are indicated. **(B)** Western blot analysis of the interaction of NRF2 with mutant p53 and p53 PA’s in co-IP assay upon overexpression of empty- (HA) or TNV p53 PA’s vectors in H1299 cells stably overexpressing p53R175H. Upon IP with the anti-NRF2 antibody, immunoprecipitated proteins (IP) and input cell lysates (INPUT) were analyzed by western blot with the indicated antibodies; size markers are indicated. **(C)** Western blot analysis of the interaction of NRF2 with mutant p53 in co-IP assay upon overexpression of empty- (0) or increasing amounts of pep1-encoding vector (15μg, 30μg, 60μg) into H1299 cells stably overexpressing p53R175H. Upon IP with anti-NRF2 antibody, immunoprecipitated proteins (IP) and input cell lysates (INPUT) were analyzed by western blot with the indicated antibodies; size markers are indicated. **(D)** Expression of selected NRF2 targets in H1299 cells transfected with empty- or p53 R175H vector (R175H) alone or in combination with mutant p53 silencing (si*TP53*) or with pep1 overexpression. Expression levels are given relative to *ACTB*. **(E,F)** Chromatin immunoprecipitation (ChIP) of NRF2-binding regions from HMOX1 (E) and TXN (F) transcription regulatory sequences using anti-p53 or anti-NRF2 antibodies upon siRNA-mediated silencing of mutant *TP53*, *NRF2* or control siRNA (siCtrl). ChIP enrichment in anti-p53 (DO-1) and anti-NRF2 antibody IP is compared to the control IgG antibody IP. Data shown in D-F are the means ± s.d. of n=3 independent experiments, ANOVA test with Bonferroni correction: ^*^ p<0.05, ^**^ p<0.01, ^***^ p<0.001.

We have previously shown that mutant p53, via its DNA-binding domain, interacts with NRF2 and cooperatively both proteins activate proteasome subunit genes’ transcription [14]. We have also hypothesized that the interaction between mutant p53 and NRF2 is crucial for the transcriptional regulation of proteasome subunit genes and possibly, of other NRF2 targets but we did not characterize in detail the region of p53 involved in NRF2 binding. To this aim we used here a previously described protein scaffold system to expose peptide aptamers in cells for protein interaction studies [25]. Six peptides of the length of 30 amino acids containing sequences corresponding to the human p53 DNA-binding domain (DBD) were expressed inside the scaffold protein fused with HA tag cloned within an eukaryotic expression vector [25] (Supplementary Figure 3D). The p53-null H1299 cancer cells were transfected with plasmids expressing the full-length mutant p53 R175H or the six peptides spanning the DBD of mutant p53 and cell lysates were subjected to immunoprecipitation of endogenous NRF2. As shown in Figure 2A among the six peptides only peptide1 (pep1) corresponding to aa 98-128 of the mutant p53 DBD was co-immunoprecipitated with NRF2. Moreover, in H1299 cells stably overexpressing full-length mutant p53, ectopic expression of the plasmid vector carrying pep1 was able to compete with mutant p53 full-length protein for binding to endogenous NRF2, as shown by co-immunoprecipitation assays with the anti-NRF2 antibody (Figure 2B). This effect increased when increasing amount of the pep1 encoding vector was used (Figure 2C).

We next verified whether this peptide was able to interfere with the mutant p53-dependent regulation of NRF2 transcriptional targets. As shown before [14], introduction of mutant p53 variant R175H in the p53-null background of H1299 cells caused the up-regulation of proteasome subunit genes (*PSMA2* and *PSMC1*) known targets of NRF2, and this effect was strongly counteracted by overexpressing pep1 (Supplementary Figure 3E).

Next, we asked if pep1 by preventing mutant p53/NRF2 interaction could affect also other NRF2 targets differentially regulated by mutant p53. In H1299 cells both transient and stable overexpression of mutant p53 R175H enhanced *TXN* and *PSMC1* gene transcription, while significantly repressed *HMOX1* expression (Figure 2D). Conversely, upon downregulation of mutant p53 levels with a specific siRNA, H1299^+mtp53R175H^ cells showed a significant up-regulation of *HMOX1* transcript and a down-modulation of *TXN* and *PSMC1* gene transcription. We also observed comparable, opposite effects on *TXN* and *PSMC1* and on *HMOX1* in mutant *TP53*-silenced H1299^+mtp53R175H^ cells and in H1299^+mtp53R175H^ cells overexpressing pep1 (Figure 2D).

These results indicate that aa 98-128 of p53 are involved in the interaction of mutant p53 with NRF2 and that expression of a peptide corresponding to that sequence is able to dissociate the complex between the two proteins with a clear impact on the transcriptional regulation of NRF2 targets by mutant p53.

### Mutant p53 presence on ARE elements in regulatory regions of NRF2 targets is required for activation of their transcription

To dissect the mechanism by which the concerted action of mutant p53 and NRF2 can either enhance or repress transcription, chromatin immunoprecipitation (ChIP) experiments were performed in MDA-MB-231 cells using NRF2 or p53 antibodies and the regulatory regions of both activated and repressed NRF2 target genes were analyzed.

We found out that in MDA-MB-231 cells NRF2 binds the regulatory elements of its targets even without externally applied oxidative stress (Figure 2E, 2F and Supplementary Figure 3F, 3G).

In these conditions mutant p53 is co-recruited on TXN and TXNRD1 gene promoters together with NRF2 (Figure 2F and Supplementary Figure 3G). Silencing of mutant *TP53* decreased NRF2 recruitment to these regions, while silencing of *NRF2* completely abolished mutant p53 binding indicating that mutant p53 requires NRF2 for binding ARE sequences on activated gene promoters, an effect that we have previously shown also for proteasome subunit genes [14]. On the other hand we did not find mutant p53 binding to the promoter of ABCC3, nor to an ARE-containing distant enhancer (EN2) of HMOX1, which is a canonical regulatory element bound and activated by NRF2 [26] (Supplementary Figure 3F and Figure 2E). Silencing of mutant *TP53* increased the efficiency of NRF2 binding to the HMOX1 enhancer (Figure 2E).

In normal cells under unstressed conditions NRF2 resides mainly within the cytoplasm, while in cancer cells, the intracellular chronic oxidative stress causes the accumulation of a fraction of NRF2 in the nucleus in the absence of external stress stimuli [14, 27]. Interestingly, we observed that overexpression of two different GOF mutant p53 variants in H1299 induced an increase in nuclear NRF2 (Supplementary Figure 4A, 4B) which was comparable to that observed upon oxidative stress induction (Supplementary Figures 2A and 4C). Moreover, when we silenced mutant *TP53* expression in MDA-MB-231 or in MCF10A overexpressing mutant p53 R175H, we observed that the majority of NRF2 was localized in the cytoplasm (Supplementary Figure 4D, 4E).

These results suggest that mutant p53 increases NRF2 localization to the nucleus of cancer cells where it redirects NRF2 to ARE elements of specific genes, activating their transcription (*TXN*, *TXNRD1)*, and conversely it sequesters NRF2 from other targets (*HMOX1*, *ABCC3*) leading to their downregulation.

### Cancer cell survival and migration under oxidative stress relies on the mutant p53-depedent differential regulation of NRF2 targets

**Figure 3:**
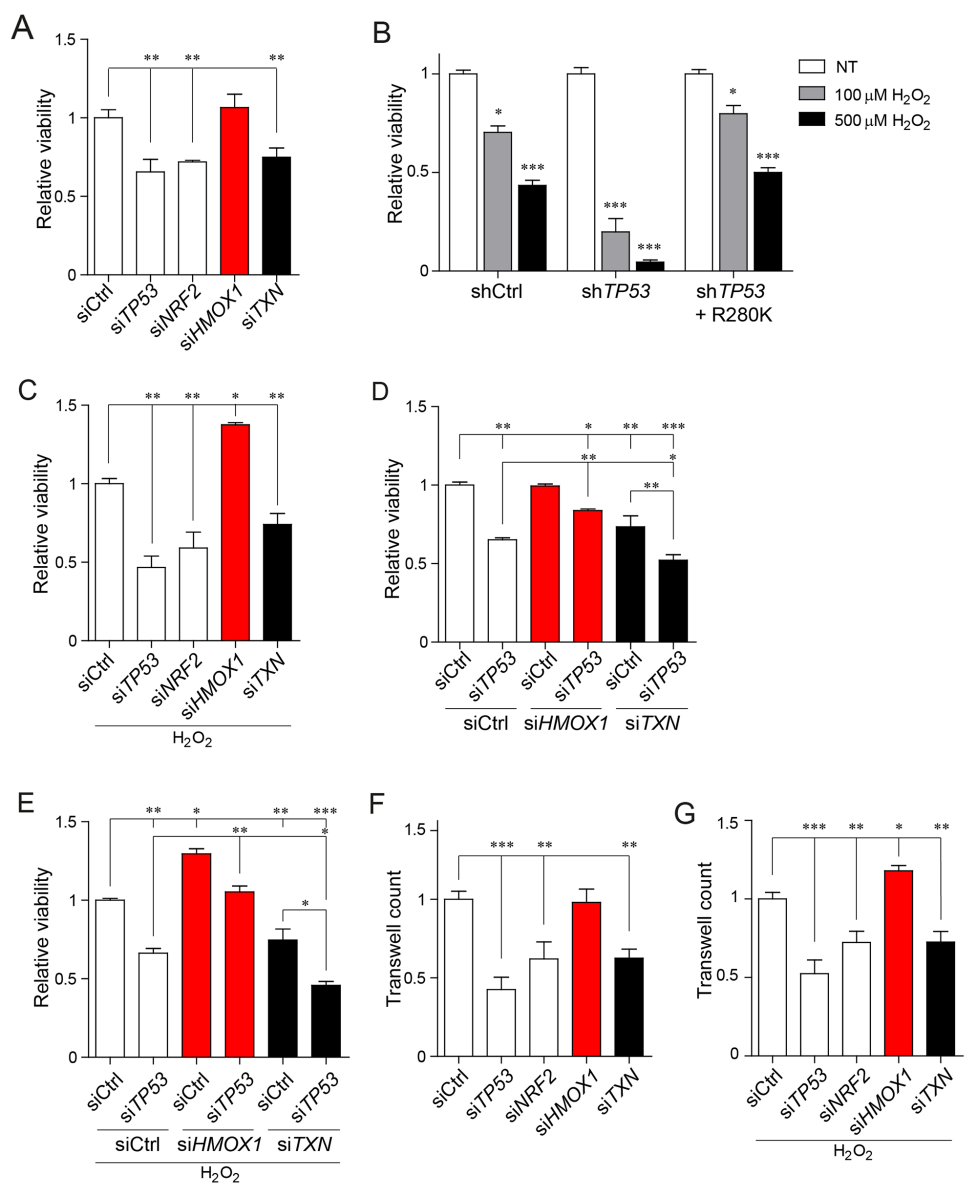
Cancer cell viability and migration under oxidative stress rely on the mutant p53-depedent differential regulation of NRF2 targets **(A)** Viability measurement of MDA-MB-231 cells upon *TP53*, *NRF2*, *TXN*, *HMOX1* or control silencing. **(B)** Viability measurement of MDA-MB-231 cells stably silenced for control (shCtrl), *TP53* (sh*TP53*), or *TP53* together with stable mutant p53 R280K overexpression (sh*TP53* + R280K) in unstressed (NT) or under oxidative stress conditions (100μM or 500μM H_2_O_2_, 48 hours treatment). **(C)** Viability measurement of MDA-MB-231 cells upon *TP53*, *NRF2*, *TXN*, *HMOX1* or control silencing under low oxidative stress conditions induced by overnight treatment with 100μM H_2_O_2_. **(D)** Viability measurement of MDA-MB-231 cells upon co-silencing of p53 and NRF2 downstream targets (*HMOX1* or *TXN*). **(E)** Viability measurement of MDA-MB-231 cells after co-silencing of p53 and NRF2 downstream targets (*HMOX1* or *TXN*) under low oxidative stress conditions induced by overnight treatment with 100μM H_2_O_2_. **(F)** Transwell migration assays of MDA-MB-231 cells upon *TP53*, *NRF2*, *TXN*, *HMOX1* or control silencing. **(G)** Transwell migration assays of MDA-MB-231 cells upon *TP53*, *NRF2*, *TXN*, *HMOX1* or control silencing under low oxidative stress conditions induced by overnight treatment with 100μM H_2_O_2_. Data shown in A-G are the means ± s.d. of n=3 independent experiments, ANOVA test with Bonferroni correction: ^*^ p<0.05, ^**^ p<0.01, ^***^ p<0.001.

H_2_O_2_ is a widely used inductor of oxidative stress that activates NRF2-dependent response mechanism [28–30] and mimics the characteristic chronic oxidative stress environment of tumor cells [31–33]. As reported by others, via H_2_O_2_ production, oxidative stress modulates tumor growth and spread [31, 34] and sub-cytotoxic levels of H_2_O_2_ in tumors differentially modulate the behavior of normal and neoplastic cells [35]. In order to address the impact of the NRF2 targets activated or repressed by mutant p53 on mutant p53-dependent cancer cells phenotype, we performed viability experiments in cancer cells either exposed or not to exogenous oxidative stress by H_2_O_2_ (Figure 3A–3E and Supplementary Figure 4F).

In normal conditions, in the absence of H_2_O_2_, silencing of either mutant *TP53* or *NRF2* significantly decreased MDA-MB-231 cell viability. Similar effects were observed upon silencing of *TXN* gene, an NRF2 target induced by mutant p53 (Figure 3A). In contrast, silencing of *HMOX1*, an NRF2 target repressed by mutant p53, had no effect on the viability of MDA-MB-231 cells (Figure 3A). Treatment of MDA-MB-231 breast cancer cells with 100μM H_2_O_2_ (low oxidative stress) induced a 30% decrease of cell viability, while upon 500μM treatment (high oxidative stress) cell viability was reduced by 70% (Supplementary Figure 4F). As expected both low and high oxidative stress activated NRF2 and its targets, albeit to a different extent (Supplementary Figure 2A, 2B). Silencing of mutant *TP53* further sensitized cells to low and high oxidative stress, but overexpression of mutant p53 in MDA-MB-231 cells, silenced for the endogenous mutant *TP53* variant R280K, rescued cell survival conferring a cytoprotective effect to cancer cells (Figure 3B). In low oxidative stress conditions, silencing of *TXN* expression further reduced cell viability, while silencing of *HMOX1* had a pro-survival effect (Figure 3C). Moreover, we observed that combining *HMOX1* and mutant *TP53* silencing counteracted the impact of the sole mutant p53 silencing on cell viability in both normal and induced oxidative stress conditions, while silencing of both mutant *TP53* and *TXN* further decreased the cell viability (Figure 3D, 3E). Under the high oxidative stress (treatment with 500μM H_2_O_2_), cancer cell viability was reduced by 90% upon silencing of either *HMOX1* or *TXN* (Supplementary Figure 4G, 4H), indicating that both antioxidant defense mechanisms have to be intact for cancer cell survival in high oxidative stress conditions.

We next wanted to evaluate the impact of *TXN* and *HMOX1* silencing also on the migration capabilities of MDA-MB-231 in both unstressed and oxidative stress conditions. As shown in Figure 3F, 3G silencing of *HMOX1* resulted in increased migration of MDA-MB-231 cells under low oxidative stress. In contrast, silencing of *TXN* resulted in a drop of the migration rate both upon exposure to low oxidative stress and under normal conditions.

Altogether these results suggest that in the low oxidative stress conditions, cancer cell survival and migration depends on selective induction of a specific oxidative stress response, namely the thioredoxin system, while keeping at low levels other antioxidant systems, such as the heme oxygenase 1 system. Our data also indicate the mutant p53/NRF2 axis as a key player in fine-tuning of this response.

### Expression of NRF2 transcriptional targets activated by mutant p53 is associated with poor prognosis and with the mutant status of p53 in breast cancer patients

**Figure 4:**
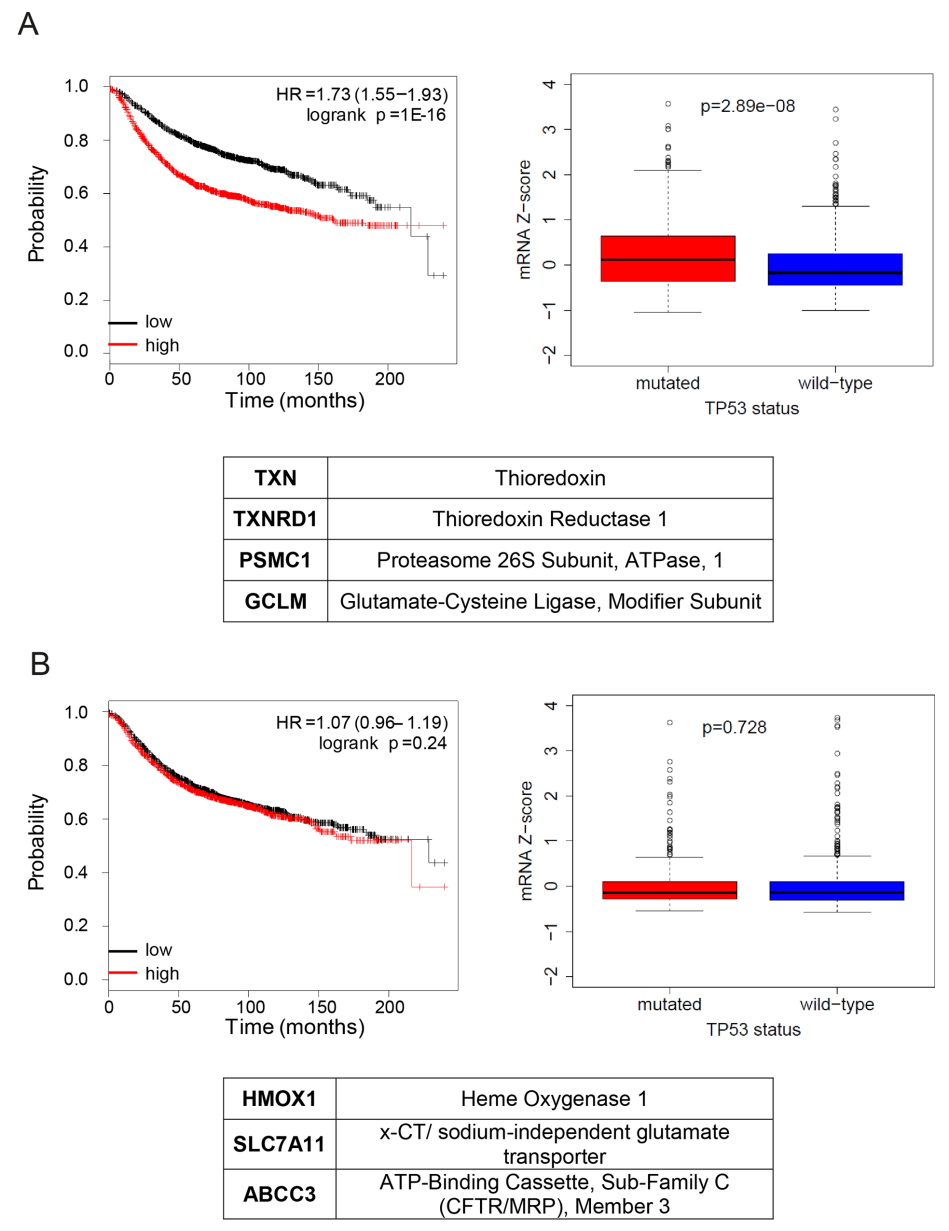
NRF2 transcriptional program activated by mutant p53 is associated with poor overall prognosis and with the mutant status of p53 in breast cancer patients **(A)** (upper panels) Left, association of the mutant p53 activated signature with a survival of breast cancer patients. The red curve (“high”) represents the survival of patients with high expression of mutant p53 activated signature, black curve (“low”) – low expression of mutant p53 activated signature. HR – hazard ratio; log-rank P – log-rank test p-value for the curves comparison. Right, association of the mutant/wt TP53 status and expression of NRF2 targets activated by mutant p53 listed in the table (*TXN, TXNRD1, GCLM, PSMC1*). Box plot: diff – difference in mean gene expression in mutant vs. wt p53 status samples; p-value is derived from Mann–Whitney *U* test. Centre represents the median, box extremes indicate the first and third quartiles, and whiskers extend to the extreme values included in the interval calculated as ±1.58 IQR/sqrt(n), where the IQR (interquartile range) is calculated as the third quartile minus the first values included in the interval calculated as ±1.58 IQR/sqrt(n), where the IQR (interquartile range) is calculated as the third quartile minus the first quartile. (lower panel) List of genes included in the signature. **(B)** (upper panels) Left, association of the mutant p53 repressed signature with survival of breast cancer patients. The red curve (“high”) represents the survival of patients with high expression of mutant p53 repressed signature, black curve (“low”) – low expression of mutant p53 repressed signature. HR – hazard ratio; log-rank P – log-rank test p-value for the curves comparison. Right, association of the mutant/wt TP53 status and expression of NRF2 targets repressed by mutant p53 (*HMOX1*, *x-CT*, *ABCC3*). Box plots: as in (A), diff – difference in mean gene expression in mutant vs wt p53 status samples; p-value is derived from Mann–Whitney *U* test). (lower panel) List of genes included in the signature.

Our data indicates a dual role of mutant p53 in regulating NRF2 transcriptional targets *in vitro*. To understand the relevance of this mechanism *in vivo*, we investigated the association of the expression levels of NRF2 targets that we found differentially regulated by mutant p53 *in vitro* with the mutant p53 status in a cohort of breast cancer patients (Figure 4). Expression of genes upregulated by mutant p53 (signature 1, Figure 4A) associated with the mutant status of p53 in breast cancer patients according to the TCGA dataset. Moreover, high expression of these genes showed association with poor prognosis in breast cancer (Figure 4A), in contrast with the expression of genes downregulated by mutant p53 (signature 2, Figure 4B). Moreover, expression of signature 2 or of *NRF2* did not correlate with the mutant status of p53 in the studied cohort (Figure 4B and Supplementary Figure 5A).

### Concomitant treatment with Auranofin and APR-246 selectively kills cancer cells bearing mutant p53

Having demonstrated the relevance of NRF2 modulation by mutant p53 on the viability and migration of cancer cells, we next asked if inhibiting the mutant p53/NRF2 axis could have a synergistic killing effect on cancer cells bearing *TP53* GOF mutations. In particular, we sought whether inhibiting mutant p53 could impair the growth of mutant p53 bearing cancer cells with perturbed NRF2 activity. In order to address this question, we investigated whether silencing of *NRF2* expression would specifically sensitize mutant p53 bearing cells to treatment with APR-246, a drug that binds and converts mutant p53 into a wild type-like, active protein [36]. Indeed, in MDA-MB-231 and MCF10A^+R280K^ cells silenced for *NRF2*, treatment with APR-246 significantly reduced viability, while wt p53 bearing cells (MCF10A or MCF7) silenced for *NRF2*, were not affected by APR-246 (Supplementary Figure 5B). These results indicate that targeting of mutant p53 with APR-246 synergizes with depleting its partner protein NRF2.

We have demonstrated here that the thioredoxin system is a downstream target and effector of the mutant p53/NRF2 axis (Figures 2D, 2F, 3 and Supplementary Figure 3G). This prompted us to test the efficacy of a combined inhibition of mutant p53 and of the *TXN*/*TXNRD1* system in killing cancer cells expressing mutant p53. Of note, a specific inhibitor of thioredoxin reductase, Auranofin, has been already approved for clinical use as an antirheumatic agent [37] and clinical trials are currently evaluating its effects on various cancer types (https://clinicaltrials.gov/ct2/).

**Figure 5:**
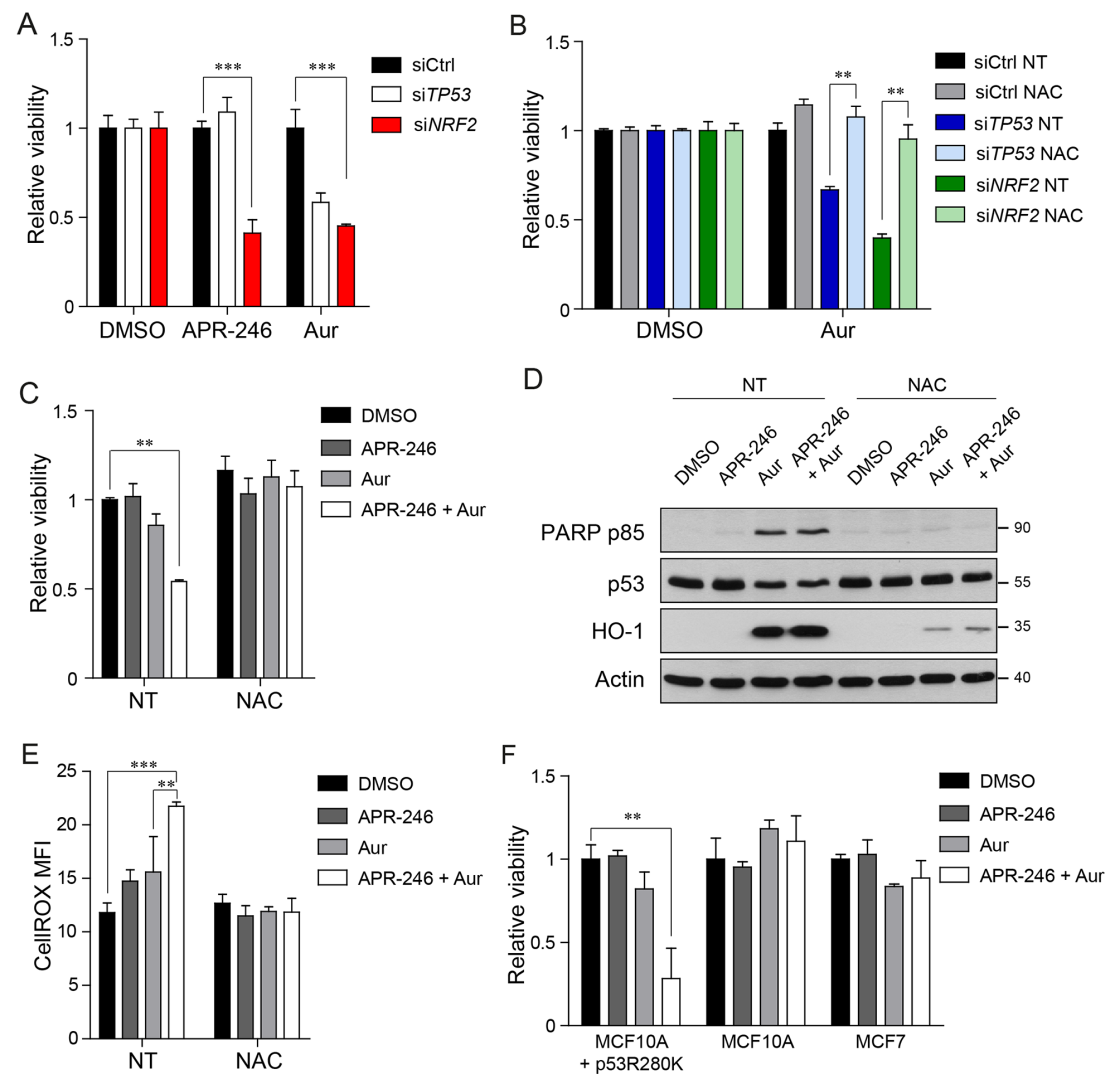
Concomitant treatment of Auranofin and APR-246 selectively kills cancer cells bearing mutant p53 **(A)** Viability measurements of MDA-MB-231 cells upon *TP53, NRF2* or control silencing and 24 hours treatment with APR-246 (25μM), Auranofin (2μM) or DMSO. **(B)** Viability measurements of MDA-MB-231 cells upon *TP53, NRF2* or control silencing and 24 hours treatment with the indicated compounds (NAC 5mM, Auranofin 2μM, DMSO). **(C)** Viability measurements of MDA-MB-231 cells upon 24 hours treatment with upon 24 hours treatment with the indicated compounds (APR-246 25μM, Auranofin 2μM, NAC 5mM, DMSO). **(D)** Western blot analysis of the apoptosis marker PARP p85 in cell lysates of MDA-MB-231 cells treated as in (C). Representative of 2 biological replicates is shown. Actin levels are reported as loading control; size markers are indicated. **(E)** CellROX mean fluorescence intensity (MFI) of MDA-MB-231 cells treated as in (C). **(F)** Viability measurements of MCF10A cells overexpressing p53R280K (MCF10A +p53R280K), MCF10A cells, and MCF7 cells respectively. Data shown in A-C, E, F are the means ± s.d. of n=3 independent experiments, ANOVA test with Bonferroni correction: ^*^ p<0.05, ^**^ p<0.01, ^***^ p<0.001.

We treated MDA-MB-231 cells silenced for mutant *TP53*, *NRF2* or control siRNA with 2μM Auranofin. Upon treatment, cells showed a significant viability decrease in mutant *TP53* and in *NRF2* silencing conditions (Figure 5A). In mutant *TP53* and in *NRF2* silenced cells the viability decrease caused by Auranofin was rescued by adding N-acetylcysteine (NAC), a compound which restores the antioxidant potential of cells [9] (Figure 5B), indicating that the viability decrease is caused by defective ROS neutralization. This result suggested that, in cancer cells silenced for mutant *TP53* or *NRF2*, Auranofin causes the viability decrease by inducing high levels of intracellular ROS the cells can not cope with, due to impairment of the mechanism activating the required antioxidant systems.

Hence, we next investigated if combining inhibition of mutant p53 by APR-246 and inhibition of the thioredoxin system by Auranofin could cause a specific lethality of mutant p53 bearing cancer cells. As shown in Figure 5C the co-treatment led to a significant down-regulation of MDA-MB-231 cell viability and enhanced apoptosis, as indicated by the accumulation of PARP p85 fragment (Figure 5D). In these conditions we also observed increased protein levels of HO-1 and a decrease in Trx protein levels (Figure 5C and Supplementary Figure 5C). We next assessed the production of ROS in response to the drug treatment by CellROX FACS analysis. The combination treatment with Auranofin and APR-246 induced significantly higher accumulation of ROS with respect to control cells and to cells treated with either APR-246 or Auranofin (Figure 5E), further indicating that the increased production of ROS levels beyond a manageable threshold could be the reason why combining treatments produces synergistic effects in killing cancer cells bearing mutant p53. Accordingly, we observed that the viability decrease and the apoptosis induction caused by the combination treatment were rescued by NAC addition (Figure 5C–5E).

A synergistic effect of the Auranofin/APR-246 treatment was observed also in MCF10A^+mtp53R280K^, while cells bearing wtp53, like MCF10A or MCF7, were not significantly affected by the drug combination in the investigated concentrations (Figure 5F).

## DISCUSSION

In normal cells, the transcription factor NRF2 is a key regulator of antioxidant pathways acting as a defense mechanism against oxidative stress. However, NRF2 activity was shown to be essential also for the survival of cancer cells by protecting them from the oxidative environment that characterizes tumors [1]. Indeed, NRF2 has been shown to be activated by oncogenic signaling, resulting in enhanced cytoprotection of cancer cells [5].

We demonstrate here that missense mutant p53 is a key interactor of NRF2 and a modulator of its transcriptional program, through which it selectively promotes a specific pro-survival oxidative stress response in cancer cells.

While NRF2-regulated transcripts, including diverse components of the oxidative stress response, are all activated upon introduction of activated Ras and Myc into breast epithelial cells, mutant p53 leads to upregulation of thioredoxin (*TXN*) and proteasome (*PSM*) systems, but to repression of heme oxygenase 1 gene (*HMOX1*) among other NRF2 targets. The difference with other oncoproteins known to activate NRF2 is likely due to the fact that while Ras or Myc family proteins primarily activate NRF2 transcription directly [4, 5], mutant p53 modulates its activity via protein interaction [14, 38] and this binding is required for regulating activation and repression of specific NRF2 targets by mutant p53. We mapped the region of p53 involved in the binding to NRF2 into the initial region of the p53’s DNA-binding domain (98-128aa). Of note, this region, which has been shown to be structurally affected in multiple mutant p53 variants, is bound by APR-246 [39], a drug inhibiting mutant p53’s gain-of-function and reactivating p53 wild-type conformation and properties [36]. The localization of the mutant p53’s region interacting with NRF2 in this domain could explain the observed binding of NRF2 to several missense p53 mutant variants and its disruption by APR-246 [14].

**Figure 6:**
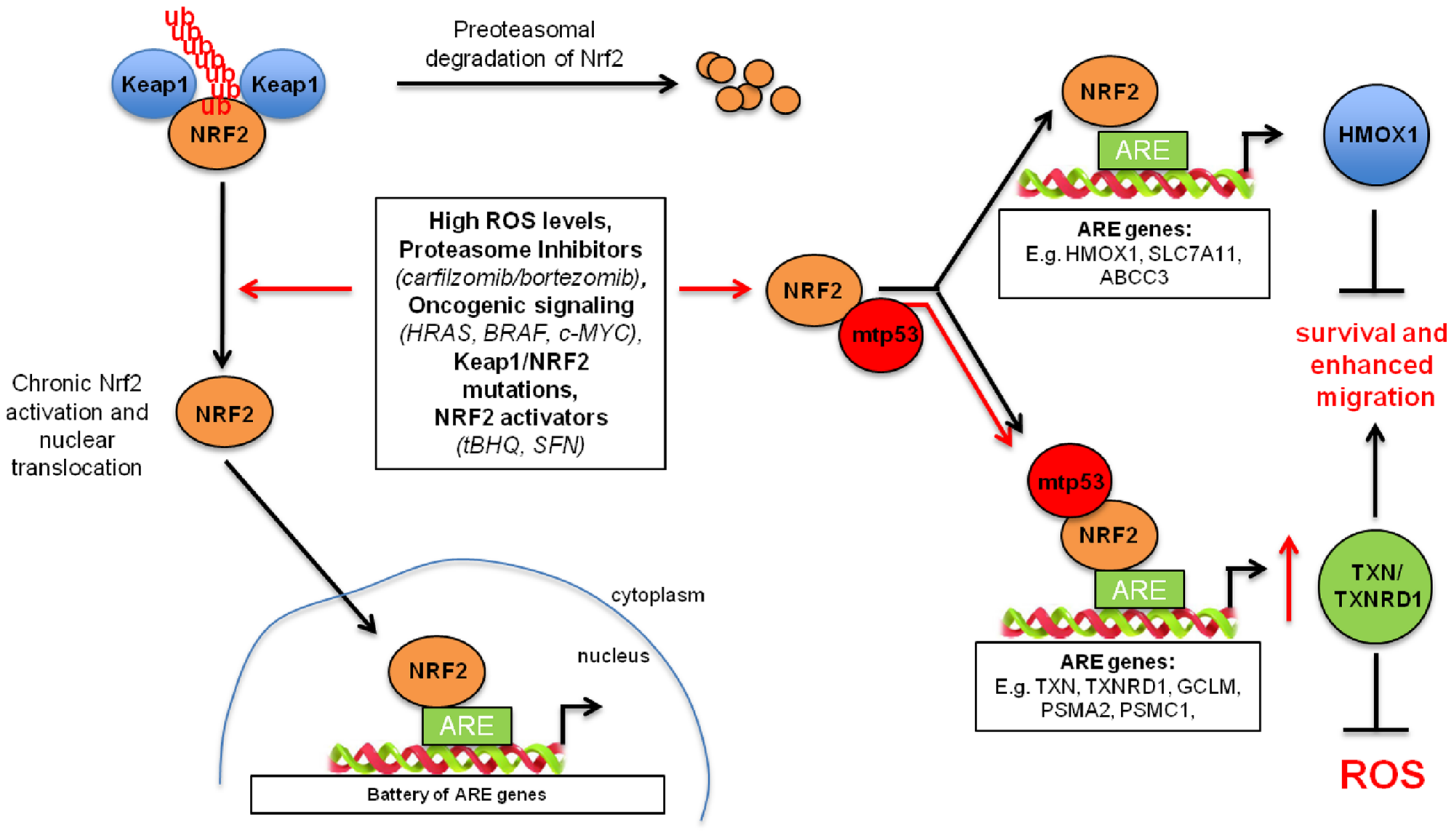
Model representing mutant p53-mediated tuning of the NRF2-dependent antioxidant pathway Left, in normal conditions, NRF2 is bound by its inhibitor Keap1 and it is directed for proteasomal degradation. Upon activation (e.g. oncogenes, oxidative stress), NRF2 translocates into the nucleus where it binds to ARE (antioxidant responsive element) sequences within promoters of target genes. Right, mutant p53, when expressed, selectively activates NRF2 downstream targets, thioredoxin and proteasome systems. This effects in acquiring the resistance to microenvironmental stress. At the same time mutant p53 by increasing the presence of NRF2 on the gene promoters of TXN or TXNRD1 sequesters NRF2 from canonical NRF2 regulatory elements of other NRF2 targets (e.g. HMOX1).

Our results indicate that mutant p53 binding to NRF2 leads to an increased nuclear localization of NRF2 on ARE-containing regulatory sequences, leading to specific effects on NRF2 targets’ transcription, resulting in the transcriptional activation of genes such as *TXN* and the proteasome encoding genes, and in the repression of others such as *HMOX1* and *ABCC3* (Figure 6). In addition, our experiments suggest that NRF2 targets can be divided in two categories. The first category consists of NRF2 inducible genes whose expression is low in the absence of oxidative stress (*ABCC3* and *HMOX1*; Supplementary Figures 2D and 5C). The other group consists of basally active NRF2 targets whose expression is already high in the same conditions (*TXN* and *TXNRD1*; Supplementary Figures 2D and 5C). Consistently, our ChIP results indicate that the binding of NRF2 to the regulatory elements of its basal targets is much higher than the binding measured for inducible targets. Our results indicate that mutant p53 might selectively contribute to repress the inducible NRF2 targets and cooperate to activate the basal ones.

The biological effect of the mutant p53/NRF2 interplay is an increased survival of cancer cells under oxidative stress. The presence of increased ROS levels is a known feature of tumor microenvironment [40, 41] and induction of the thioredoxin/thioredoxin reductase (*TXN/TXNRD1)* system is known to be beneficial for the survival of cancer cells [9, 42]. Indeed *TXN* is a critical NRF2 target and belongs to the NRF2 target signature activated by mutant p53, whose increased expression is associated with poor prognosis in breast cancer. Accordingly, high expression of *TXN* and *TXNRD1* has been previously associated to high grade breast cancers [43]. Consistently, we show here that silencing of *TXN* strongly decreases the survival and migration of breast cancer cells under oxidative stress. In contrast, silencing of *HMOX1* has opposite effects. In agreement, several studies suggest that, while having cytoprotective functions in untransformed cells, *HMOX1* plays a cytotoxic role in cancer cells [44, 45].

Overall our results shed new light onto the complex role of antioxidant systems in cancer initiation and progression. Increased production of ROS is essential to enable and sustain a highly metastatic phenotype [46]. However by preventing an excess of damage due to ROS accumulation, antioxidant systems such as thioredoxin or glutathione are also required for cancer initiation, and inhibition of these ROS scavenging mechanisms, combined with pro-oxidizing agents, seems to be effective in the treatment of chemoresistant tumors [9, 40, 41, 47, 48].

On the basis of our observations, we tested a combinations of drugs aimed at blocking the biological effects exerted by the mutant p53-NRF2 axis in cancer cells. Our results indicate that breast cancer cells expressing mutant p53 can be efficiently eliminated by combining the two drugs APR-246 and Auranofin. The first one is a covalent inhibitor of mutant p53 that was also described to affect the cellular redox status by targeting selenoprotein thioredoxin reductase 1 [49], while Auranofin is a known inhibitor of the thioredoxin system [50]. Recently, multiple reports have indicated that targeting the thioredoxin system with various drugs could be an efficient strategy for killing chemoresistant cancer cells [9, 37, 50–52]. Indeed, Auranofin and APR-246 in combination turned out to synergistically affect cancer cell survival by blocking mutant p53-depentend antioxidant systems both directly and indirectly. Interestingly, in a recent study mutant p53 was shown to mediate the repression of the NRF2 target gene *SLC7A11* and a concomitant use of APR-246 with a glutathione synthesis inhibitor was revealed as an approach to eliminate cancer cells with mutant p53 [38]. This result suggests that understanding of the mutant p53-depedent tuning of the NRF2 program may result in several efficient anti-cancer treatment strategies.

Our work revealed the existence of a mutant p53/NRF2 axis that specifically exploits the thioredoxin system to sustain the survival of breast cancer cells under oxidative stress. These findings provide important advances in the understanding of NRF2 regulation in cancer and open up new therapeutic opportunities for breast cancers expressing mutant p53.

## MATERIALS AND METHODS

### Cell lines and treatments

All cell lines were purchased from ATCC. Human cell lines MDA-MB-231 (p53 R280K), MDA-MB-468 (p53 R273H) were cultured in DMEM medium (BioWhittaker) supplemented with 10% FCS (ECS0180L, Euroclone), and antibiotics (DE17-602E, Lonza). BT-549 (p53 R249S), H1299 (p53-null) cells were cultured in RPMI medium (BioWhittaker) supplemented with 10% FBS and antibiotics. SUM-149 (p53 M237I) cells were cultured in DMEM:F12 Ham’s medium 1:1, supplemented with 10% FCS and antibiotics. MCF7 (p53 wt) were cultured in EMEM (BioWhittaker), supplemented with 1% non-essential aminoacid solution (Sigma), 10% FBS and antibiotics. MCF10A (p53 wt, sh*TP53* and stable mutant p53 overexpressing cell lines) cells were maintained in DMEM:F12 Ham’s medium 1:1, supplemented with 5% horse serum, insulin (10 μg/ml), hydrocortisone (0.5 μg/ml) and epidermal growth factor (EGF 20 ng/ml), if needed - with addition of selection antibiotics.

All human cell lines were subjected to STR genotyping with PowerPlex 18D System and confirmed in their identity comparing the results to reference cell databases (DMSZ, ATCC, and JCRB databases). Mutant p53 cell lines have been confirmed to express indicated mutant *TP53* variants by sequencing of the full-length p53 mRNA [14]. All the cell lines have been tested by PCR/IF for the *Mycoplasma* presence. No cell lines used in this study were found in the database of commonly misidentified cell lines that is maintained by ICLAC and NCBI Biosample. H_2_O_2_ (Sigma 216763), menadione (Sigma M5625), sodium arsenite (Sigma S7400), Auranofin (Enzo Life Sciences BML-2842-0100) and NAC (N-Acetyl-L-Cysteine, Sigma A7250) were resuspended and used as indicated in the respective datasheets.

### Plasmids

pSR-shRNAp53 PuroR used to stably silence *TP53* expression was a kind gift of R. Agami. si/shRNA resistant N-terminally HA-tagged p53 constructs p53R280K and p53R175H were generated by first introducing 4 silent point mutations in the region targeted by p53 siRNA#1/shRNA by site-directed mutagenesis in pcDNA-HA-p53, subsequent introduction of missense point mutations and subcloning of sequenced p53 cds constructs to pMSCV-HA BlastR retroviral vector.

### Transfection

For retrovirus production (stable silencing of *TP53* and ectopic overexpression of mutant p53s) low confluent HEK 293GP packaging cells were transfected with appropriate vectors by calcium phosphate precipitation. After 48-72 hours the virus-containing medium was filtered and added to target cells (MDA-MB-231, MCF10A or H1299). Cells were selected with puromycin (0.5 μg/ml) and/or blasticidin (2 μg/ml).

H1299 cells were transfected using Lipofectamine 2000 reagent (Invitrogen) following the manufacturer’s instructions.

For siRNA transfections, all cells lines were transfected at 40-60% confluence with 24 hours interval (to increase efficiency of silencing), with 50 nM siRNA oligonucleotides using Lipofectamine RNAiMax (Invitrogen), following manufacturer’s instructions. After 48 hours cells were processed. siRNAs coding sequence used in this work are listed in the Supplementary Table 1.

### Total RNA extraction and RT-qPCR analysis

Total RNA was extracted with QIAzol (Qiagen) following manufacturer’s instructions. 1μg of total RNA was reverse-transcribed with QuantiTect Reverse Transcription (Qiagen). Real-time qPCR in technical duplicates from each biological replicate was performed using SsoAdvanced™SYBR Green Master Mix (Biorad) on a CFX96 Real-Time PCR System (Biorad). Expression levels are given relative to *ACTB*, *GAPDH* or histone *H3.* The list of qPCR primers used is provided in the Supplementary Table 2. Key NRF2 downstream targets analyzed in Figure 1A were selected from available datasets. For the following experiments we narrowed down the signatures to the most strongly regulated genes by mutant p53.

### ChIP

Chromatin was immunoprecipitated with p53 DO-1 (Santa Cruz) or NRF2 antibody (Abcam), as previously described [14]. As negative controls IgGs purified from rabbit or mouse serum were used. Coimmunoprecipitated DNA was analyzed by real-time PCR on a CFX96 Real-Time PCR System (Biorad), using SsoAdvanced™SYBR Green Master Mix (Biorad). Promoter occupancy was calculated as percent of input chromatin immunoprecipitated using the 2^-DCt^ method. Primer sequences are shown in the Supplementary Table 3.

### Statistics and reproducibility

The statistical analysis of experimental results is described in the figure legends, along with number of biological replicates and plotted error types (s.d. – standard deviation). Statistic tests were performed and *p*-value thresholds were obtained using GraphPad 6.0.

### Patient survival and mutation status association analysis

To verify the correlation of the gene signatures and breast cancer clinical data, survival analysis was performed on a breast cancer meta-dataset composed by 3458 samples using the Km-plotter online analysis tool [53]. In order to perform the analysis on the greatest possible number of patients, for each gene, we selected only HGU133A probe-sets. The samples were split into two groups according to median expressions of the proposed signatures. The two groups were then compared by survival analysis. The Kaplan-Maier curves of relapse free survival time (RFS), the hazard ratio with 95% confidence intervals and log-rank test p-values were calculated. For each signature we selected the top 3 or 4 genes up-regulated or down-regulated by mutant p53.

Gene expression data, *TP53* mutation status and clinical annotation for Breast Invasive Carcinoma, (TCGA datasets) have been obtained from Cancer Genomics Data Server using the cgdsr package for R (https://cran.r-project.org/web/packages/cgdsr/index.html). The datasets were chosen for analysis according to the wt *TP53* vs mutant *TP53* status availability, with *TP53*-null samples excluded. For each patient we defined the levels of mtp53 upregulated or mtp53 not upregulated signatures expression as the mean of the expression values of all the genes included in the signature. The genes composing each used signature are described in Figure 4A, 4B. The statistical differences between the distributions of expression values in the two molecular conditions (mutated *TP53* and wt *TP53*) were calculated by Mann–Whitney *U* test in R/Bioconductor environment (R Core Team, 2013).

Pearson’s Chi-squared test with Yates’ continuity correction has been performed to test independence between TP53 status and a signature expression. All statistical analysis has been performed using R statistical analysis environment.

### Western blot analysis

Total cell extracts were prepared in RIPA buffer without SDS (150mM NaCl, 50mM Tris-HCl pH8, 1mM EDTA, 1% NP-40, 0.5% Na-deoxycholate) supplemented with 1 mM PMSF, 5 mM NaF, 1 mM Na3VO4, 10μg/ml CLAP protease inhibitor cocktail (Sigma). Protein concentration was determined with Bio-Rad Protein Assay Reagent (Bio-Rad). Lysates were resolved by SDS/PAGE and transferred to nitrocellulose (Millipore). Western blot analysis was performed according to standard procedures using primary antibodies listed in Supplementary Table 4. Western blots experiments were performed in at least 2 biological replicates, the representative is shown.

### Cell fractionation

Cells were scraped in PBS and washed two times. The pellet obtained after the last centrifugation was resuspended in Cytoplasmic-buffer (10mM HEPES pH7.9, 1.5mM MgCl2, 10mM KCl, 0.5mM DTT, 0.1% NP-40) supplemented with inhibitors (1mM PMSF, 5mM NaF, 10μg/ml CLAP, 1mM Na3VO4); lysis was obtained gently pipetting a couple of times. After 3’ in ice, lysates were centrifuged at 2500g for 5’ at 4°C; the supernatant was collected as the cytoplasmic fraction. The pellet was washed twice in Wash-buffer (10mM HEPES pH7.9, 1.5mM MgCl2, 10mM KCl, 0.5mM DTT) and then Nuclei in the pellet were resuspended in Nuclear-buffer (20mM HEPES pH7.9, 1.5mM MgCl2, 420mM NaCl, 0.2mM EGTA, 0.5mM DTT, 25% glycerol) supplemented with inhibitors (1mM PMSF, 5mM NaF, 10μg/ml CLAP, 1mM Na3VO4); nuclear extract was recovered by centrifugation at 15000g for 15’ at 4°C.

### Protein interaction studies

Coimmunoprecipitation experiments with endogenous proteins were performed by lysing cells in the Co-IP buffer (NaCl 150mM, Tris-HCl pH8 50mM, EDTA 1mM, NP40 0.5%, glycerol 10%) with protease inhibitors. Samples were cleared by centrifugation for 30 min at 13000g at 4°C and incubated overnight at 4°C with the specific antibody. After 1h incubation with protein G-Sepharose (GE Healthcare), immunoprecipitates were washed three times in Co-IP buffer, resuspended in a sample buffer, and analyzed by western blotting. For Co-IP of endogenous p53 or NRF2, DO-1 (sc-126, Santa Cruz) and EP1808Y (ab62352, Abcam) primary antibodies were used respectively, and mouse or rabbit normal IgGs (Santa Cruz) as negative controls.

### Immunofluorescence

Cells were fixed in 4% paraformaldehyde for 20 min, washed with PBS, permeabilized with Tryton 0.1% for 5 min and blocked in PBS + FBS 3% for 30 min. Antigen recognition was done by incubating primary antibodies against p53 and NRF2 for 1hr at 37°C, followed by incubation with AlexaFluor 568 and 488 conjugated secondary antibodies. Nuclei were counterstained with Hoechst 33342 (LifeTechnologies). Representative fluorescence images of 3 biological replicates were taken with a x 630 magnification on a Leica DM4000B microscope equipped with a Leica DFC420C camera and acquired with Leica Application Suite 2.5.0 R1 (Leica Microsystems).

### Migration assay

Migration assay were performed by seeding cells at a density of 5,000 cells per well in 24-well PET inserts (8.0 mm pore size, Falcon). After 16 hr, cells that passed through the filter were fixed in 4% PFA, stained with 0.5% crystal violet and counted with a x 20 objective on CK30 Olympus optical microscope. Results shown are the average of 6 fields of view of 6 separate filters for each experimental condition.

### ROS detection

For CellROX analysis, CellROX^®^ Green Reagent (Thermo Fisher Scientific) was used as previously described [54]. Analyses were performed on a FACSCalibur cell sorter (Becton Dickinson) and data were analyzed with FlowJo software for Mac (FlowJo, LLC 2013-2016).

### Viability assay

6-10x10^4^ cells were plated in 96-well plates (white, transparent bottom), after 24 hours they were treated as indicated in figures and assayed for viability using ATPlite™ OneStep reagent (Perkin Elmer), according to the manufacturer’s instructions. Luminescence intensity was measured using EnSpire plate fluorometer (Perkin Elmer). 3 biological replicates were performed, for each experiments means from 3 technical replicates (3 wells) were used.

## SUPPLEMENTARY MATERIALS FIGURES AND TABLES


