# Supplementary material for: Mutant p53 tunes the NRF2-dependent antioxidant response to support survival of cancer cells
Source: Oncotarget. 2018 Apr 17;9(29):20508–23. doi: 10.18632/oncotarget.24974 (PMC5945496; doi:10.18632/oncotarget.24974)
Supplement: Supplementary file 1 [file oncotarget-09-20508-s001.pdf]

Mutant p53 tunes the NRF2-dependent antioxidant response to support survival of cancer cells

SUPPLEMENTARY MATERIALS

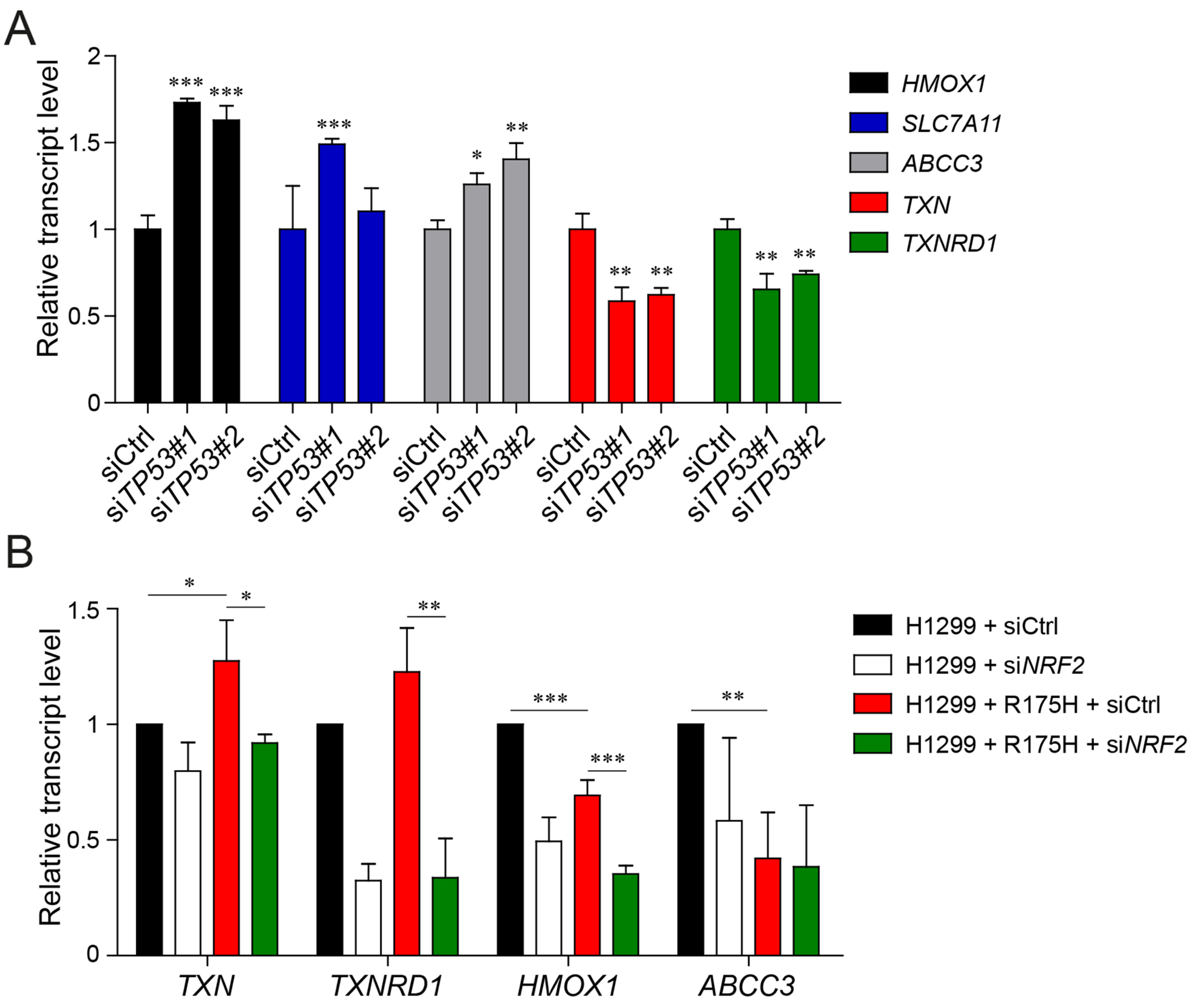

Supplementary Figure 1: (A) Expression of selected NRF2 targets in MDA-MB-231 cells upon control- or two different *TP53* silencing (sh*TP53#1,* sh*TP53#2*) (Means of n=3 independent experiments, s. d. are shown, ANOVA test with Bonferroni correction: *p<0.05, **p<0.01, ***p<0.001). **(B)** Expression of selected NRF2 targets in H1299 cells stably over-expressing empty- or mutant p53 R175H vector (R175H) alone or in combination with NRF2 silencing (si*NRF2*) (Means of n=3 independent experiments, s.d. are shown, ANOVA test with Bonferroni correction: *p<0.05, **p<0.01, ***p<0.001).

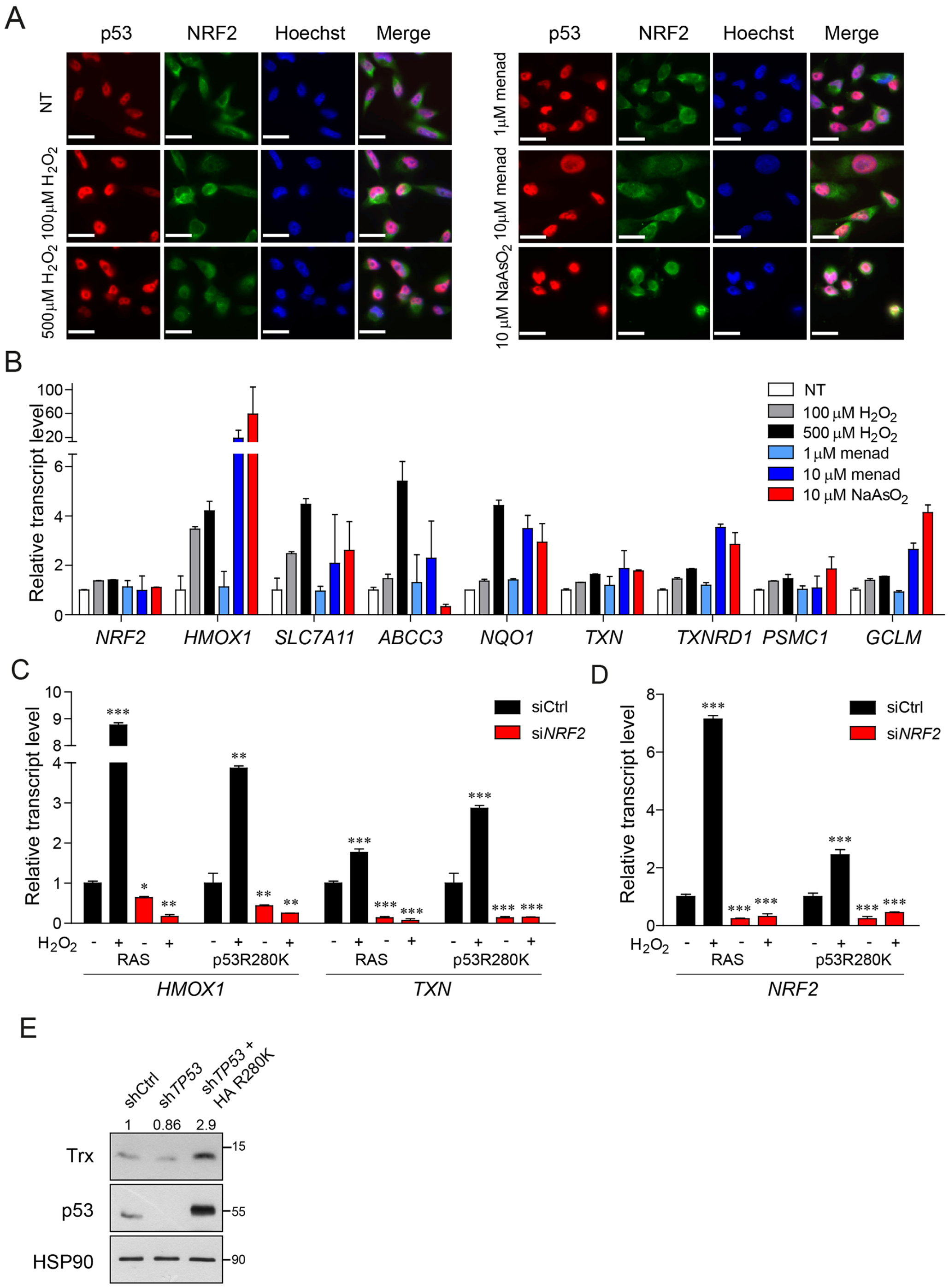

Supplementary Figure 2: **(A)** Representative fluorescence microscope images of p53 (red), NRF2 (green) and Hoechst (blue) staining in MDA-MB-231 cells in untreated (NT) condition or upon 24h treatment with the indicated compounds. All immunofluorescence results are representatives of 3 biological replicates. Scale bar 50 μm. **(B)** Expression of NRF2 targets in MDA-MB-231 cells in response to 48h treatment with the indicated compounds or not treated (NT). (Means of n=3 independent experiments, s.d. are shown). **(C)** Expression of *HMOX1* and *TXN* in MCF10A cells overexpressing the oncogenic variant H-Ras G12V (RAS) or in MCF10A cells stably silenced for wt p53 and overexpressing mutant p53 (p53R280K) upon *NRF2* or control silencing in both unstressed (−) and oxidative stress conditions (100μM H_2O_2) (Means of n=3 independent experiments, s.d. are shown, ANOVA test with Bonferroni correction: $^{*}p<0.05$, $^{**}p<0.01$, $^{***}p<0.001$). **(D)** Same as in (C) for *NRF2* transcript levels (Means of n=3 independent experiments, s.d. are shown, ANOVA test with Bonferroni correction: $^{*}p<0.05$, $^{**}p<0.01$, $^{***}p<0.001$). **(E)** Western blot analysis of the indicated protein in MDA-MB-231 cells stably silenced for control (shCtrl), *TP53* (sh*TP53*), or *TP53* together with stable mutant p53 R280K overexpression (sh*TP53* + HA R280K). HSP90 levels are reported as loading control; size markers are indicated. Protein quantification was obtained with ImageJ software, protein levels were normalized to HSP90.

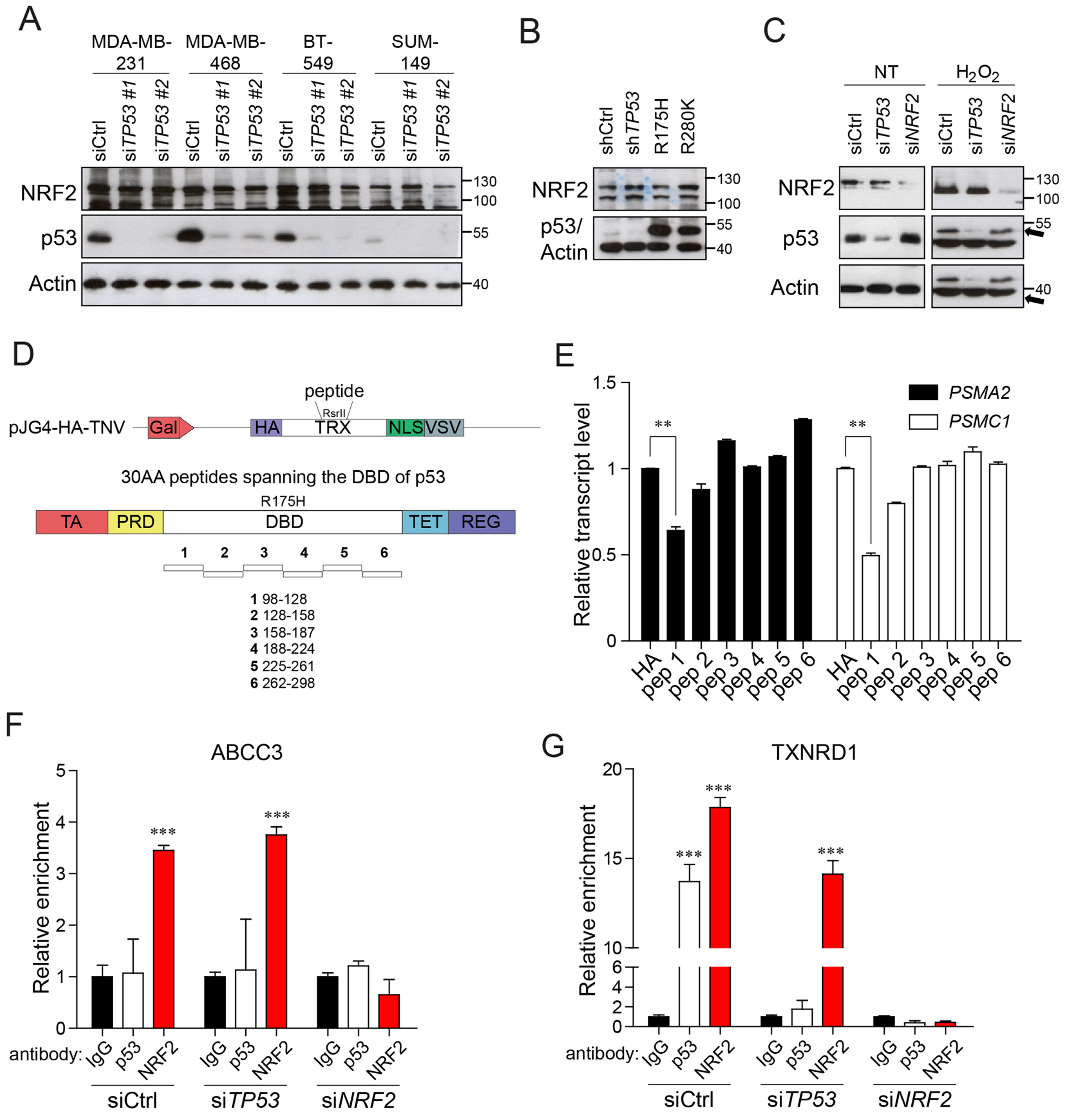

Supplementary Figure 3: (A) Western blot analysis of the indicated proteins in cell lysates of MDA-MB-231, MDA-MB-468, BT-549, SUM-149 cells upon control- or two different *TP53* silencing (representatives of at least 2 repetitions is shown). Actin levels are reported as loading control; size markers are indicated. **(B)** Western blot analysis of the indicated proteins in cell lysates of MCF10A cells upon control- or stable *TP53* silencing and overexpression of mutant p53 R175H or R280K (representatives of at least 2 repetitions is shown). Actin levels are reported as loading control; size markers are indicated. **(C)** Western blot analysis of the indicated proteins in cell lysates of MDA-MB-231 cells upon *TP53, NRF2* or control silencing in untreated (NT) or upon treatment with 100µM H_2O_2. Actin levels are reported as loading control; size markers are indicated; corresponding bands are indicated by arrows. **(D)** Top, schematic representation of the HA-TNV vector used for peptide aptamers expression. This vector contains an expression TNV cassette, where the bacterial Trx (thioredoxin) sequence is fused with an HA tag and nuclear localization sequence (NLS). Bottom, schematic representation of human p53 and of the six peptide aptamers (PAs 1-6) spanning its DBD. **(E)** Expression of *PSMA2* and *PSMC1* in H1299 cells stably overexpressing mutant p53 variant R175H and transfected with empty- (HA) or with TNV PA's (1-6). (Means of n=3 independent experiments, s.d. are shown, ANOVA test with Bonferroni correction: $^{*}p<0.05$, $^{**}p<0.01$, $^{***}p<0.001$). **(F,G)** Chromatin immunoprecipitation (ChIP) of NRF2-binding regions from ABCC3 (F) and TXNRD1 (G) transcription regulatory sequences using anti-p53 or anti-NRF2 antibodies upon siRNA-mediated silencing of mutant *TP53*, *NRF2* or control siRNA (siCtrl). ChIP enrichment in anti-p53 (DO-1) and anti-NRF2 antibody IP is compared to the control IgG antibody IP (Means of n=3 independent experiments, s.d. are shown; ANOVA test with Bonferroni correction: $^{*}p<0.05$, $^{**}p<0.01$, $^{***}p<0.001$).

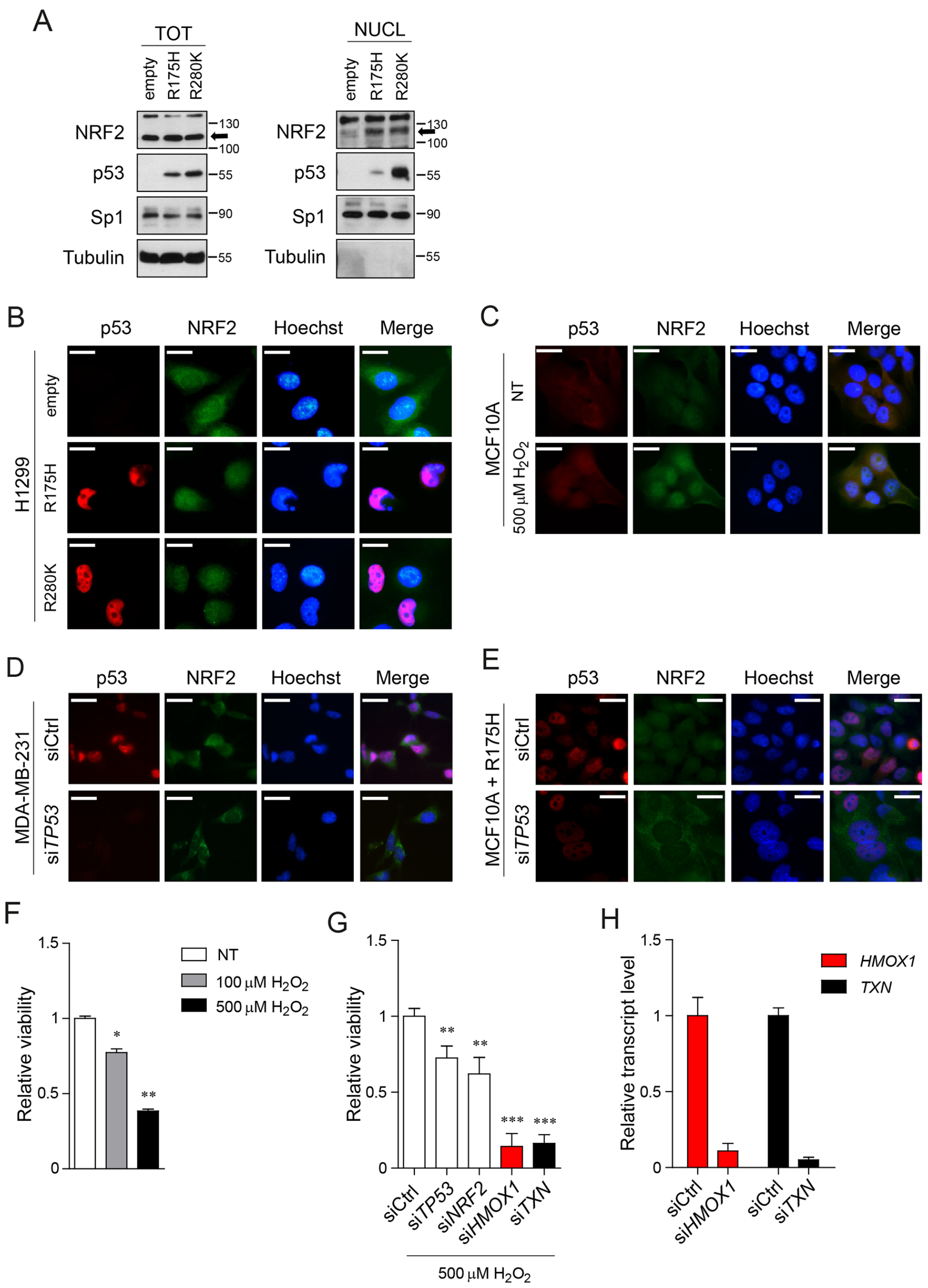

Supplementary Figure 4: (A) Left, western blot analysis of the indicated proteins in total cell lysates of H1299 cells upon stable overexpression of empty- or mutant p53 R175H or R280K vectors; right, western blot analysis of cells as in (A) that were subjected to biochemical fractionation to separate cytoplasm and nuclei, nuclear fraction is shown. Tubulin (cytoplasmic) and Sp1 (nuclear) were blotted as controls; size markers are indicated (representatives of 3 repetitions is shown). **(B-E)** Representative fluorescence microscope images of p53 (red), NRF2 (green) and Hoechst (blue) staining in: (B) H1299 cells overexpressing empty- or mutant p53 R175H and R280K vectors; (C) MCF10A cells in untreated (NT) or upon 500µM H_2O_2 treatment; (D) MDA-MB-231 cells upon *TP53* or control silencing; (E) MCF10A cells with stable overexpression of mutant p53R175H and upon *TP53* or control silencing. All immunofluorescence results are representatives of 3 biological replicates. Scale bar 50 µm. **(F)** Viability measurement of MDA-MB-231 in unstressed (NT) or under oxidative stress conditions (48h treatment with 100µM or 500µM H_2O_2) (Means of n=3 independent experiments, s.d. are shown; ANOVA test with Bonferroni correction: $^{*}p<0.05$, $^{**}p<0.01$, $^{***}p<0.001$). **(G)** Viability measurement of MDA-MB-231 cells upon *TP53*, *NRF2*, *TXN*, *HMOX1* or control silencing under mild oxidative stress conditions induced by overnight treatment with 500µM H_2O_2 (Means of n=3 independent experiments, s.d. are shown; ANOVA test with Bonferroni correction: $^{*}p<0.05$, $^{**}p<0.01$, $^{***}p<0.001$). **(H)** Expression of *HMOX1* and *TXN* in MDA-MB-231 cells after *HMOX1* or *TXN* silencing after induction of an oxidative stress with 100µM H_2O_2 (Means of n=3 independent experiments, s.d. are shown; ANOVA test with Bonferroni correction: $^{*}p<0.05$, $^{**}p<0.01$, $^{***}p<0.001$).

Supplementary Table 1: siRNA sequences

Gene name	siRNA sequence
Control siRNA	All star negative control (1027281, Qiagen)
TP53#1	GACUCCAGUGGUAAUCUUAC
TP53#2	GGUGAACCUUAGUACCUAA
NRF2 (NFE2L2)	GCAUUGGAGUGUCAGUAUG
HMOX1	GGCAGAGAAUGCUGAGUUC
TXN	ACCGUCUCAUGUCUGAAUA

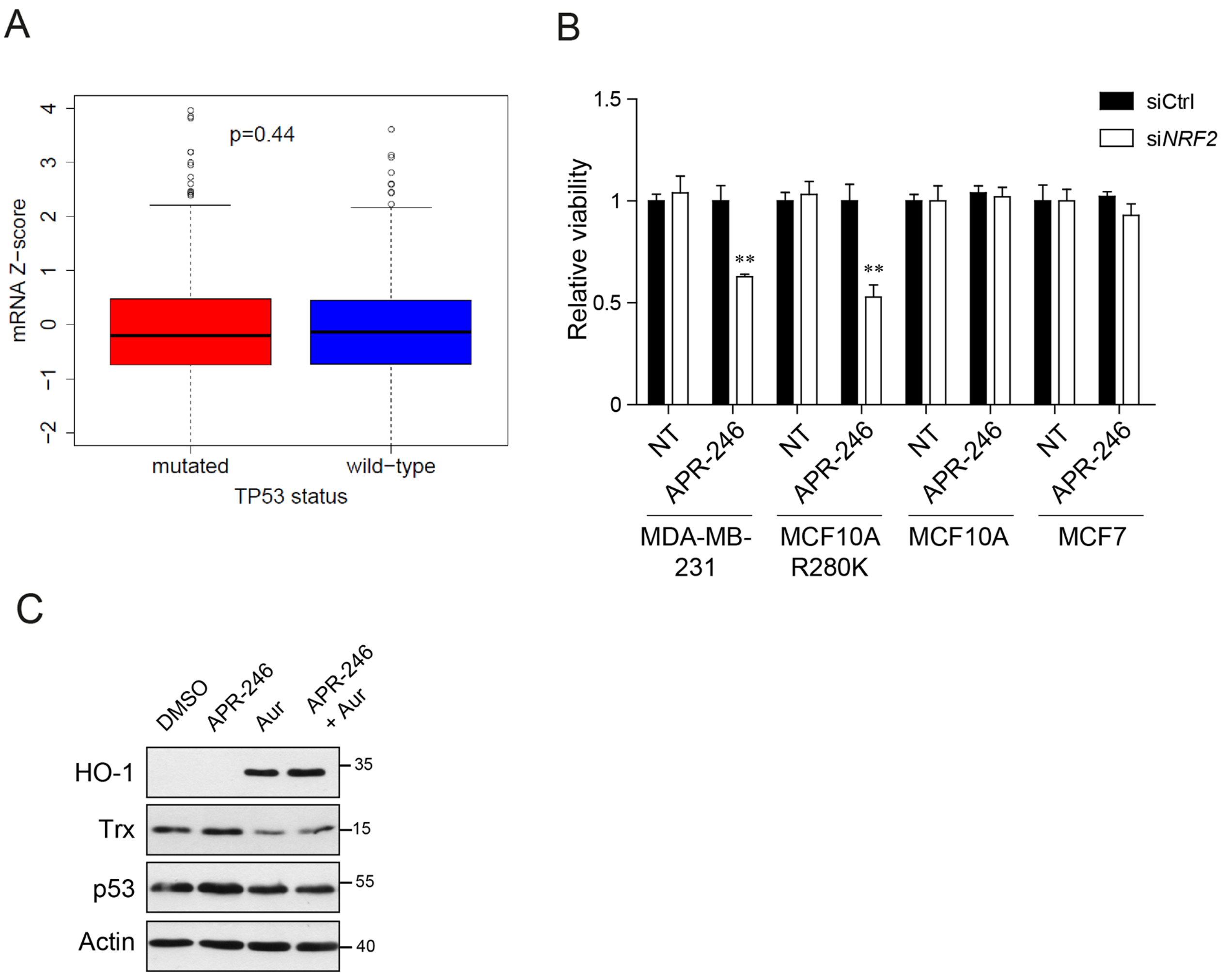

Supplementary Figure 5: (A) Right, association of the mutant/wt TP53 status and expression of *NRF2*. Box plot: diff – difference in mean gene expression in mutant vs. wt p53 status samples; p-value is derived from Mann–Whitney *U* test. Centre represents the median, box extremes indicate the first and third quartiles, and whiskers extend to the extreme values included in the interval calculated as ±1.58 IQR/sqrt(n), where the IQR (interquartile range) is calculated as the third quartile minus the first values included in the interval calculated as ±1.58 IQR/sqrt(n), where the IQR (interquartile range) is calculated as the third quartile minus the first quartile. **(B)** Viability measurement of MDA-MB-231, MCF10A with stable overexpression of mutant p53 R280K, MCF10A, and MCF7 cells silenced for *NRF2* or control (siCtrl), after overnight incubation with 25μM APR-246 or untreated (NT) (Means of n=3 independent experiments, s.d. are shown; ANOVA test with Bonferroni correction: $^{*}p<0.05$, $^{**}p<0.01$, $^{***}p<0.001$). **(C)** Western blot analysis of the indicated proteins in cell lysates of MDA-MB-231 cells upon 24h treatment with APR-246 (25 μM), Auranofin (2μM), their combination or left untreated (DMSO). Actin levels are reported as loading control; size markers are indicated.

Supplementary Table 2: Oligonucleotides for quantitative real time PCR

Gene Name	Primer sequence	Direction
NRF2 (NFE2L2)	TCCATTCCTGAGTTACAGTGTCT	FW
NRF2 (NFE2L2)	TGGCTTCTGGACTTGGAACC	REV
ACTIN	CGCCGCCAGCTCACCATG	FW
ACTIN	CACGATGGAGGGGAAGACGG	REV
H3	GTGAAGAAACCTCATCGTTACAGGCCTGGT	FW
H3	CTGCAAAGCACCAATAGCTGCACTCTGGAA	REV
GAPDH	CATGCCATCACTGCCACCC	FW
GAPDH	ACCTGGTCCTCAGTGTAGC	REV
NQO1	ATGTATGACAAAGGACCCTTCC	FW
NQO1	TCCCTTGCAGAGAGTACATGG	REV
HO-1	AACTTTCAGAAGGGCCAGGT	FW
HO-1	CTGGGCTCTCCTTGTTGC	REV
TXN	CTTGGACGCTGCAGGTGATA	FW
TXN	TCTGAAGCAACATCCTGACAGT	REV
TXNRD1	CGTCTCGGGGAAGGGAAGAT	FW
TXNRD1	CCGACCGTCCTCACATGAC	REV
GCLM	TCAACCCAGATTTGGTCAGGGAGT	FW
GCLM	TCCAGCTGTGCAACTCCAAGGA	REV
GCLC	ATGGAGGTGCAATTAACAGAC	FW
GCLC	ACTGCATTGCCACCTTTGCA	REV
ABCC3	CAGAGAAGGTGCAGGTGACA	FW
ABCC3	CTAAAGCAGCATAGACGCCC	REV
ABCC5	TGACGGAAATCGTGCGGTCTTGGT	FW
ABCC5	TGGGACCAACAGGCTTCCTGGG	REV
SLC7A11	TGGACGGTGTGTGGGGTCCT	FW
SLC7A11	CAGCAGTAGCTGCAGGGCGTA	REV
PSAT1	ACTTCCTGTCCAAGCCAGTGGA	FW
PSAT1	CTGCACCTTGTATTCCAGGACC	REV
PSMC1	TGGAGCTTCCTCTCACCCAT	FW
PSMC1	TGGCTGAGGTTTGGTTTGCT	REV
PSMA2	GTGCTTTGGCTCTTCGGGTA	FW
PSMA2	GCTTTAATTCCCACGGACGG	REV
PSMD10	AGCAGCCAAGGGTAACTTGAA	FW
PSMD10	ACTCTCTCCTCATCACAGGCT	REV

Supplementary Table 3: Oligonucleotides for quantitative real time PCR of ChIP experiments

Gene Name	Primer sequence	Direction
ChIP: TXN	ACAGTCACCCCCAGAGACAC	FW
ChIP: TXN	TCACGGTAAATTCCGGAGAG	REV
ChIP:TXNRD1	ACCCAATTAGGAGCTCTCAGC	FW
ChIP:TXNRD1	GGCTAACTGCCAGAGTCAGAA	REV
ChIP: HMOX1 (EN2)	CACGGTCCCGAGGTCTATT	FW
ChIP: HMOX1 (EN2)	TAGACCGTGACTCAGCGAAA	REV
ChIP: ABCC3	CTGAGGGCCAGAGAGATACG	FW
ChIP:ABCC3	TCTCATTTCACCCCCAACTC	REV

Supplementary Table 4: Antibodies used for Western blot, immunofluorescence, immunoprecipitation and ChIP experiments

Target protein name	ID number, producer	WB Dilution
p53 DO-1	sc-126, Santa Cruz	1:2000
NRF2 (NFE2L2)	ab62352, Abcam	1:500
Actin	A2066, Sigma	1:2000
PARP p85	G734A, Promega	1:500
Trx/TXN	sc20146, Santa Cruz	1:500
HO-1/HMOX1	ADI-OSA-110, Enzo	1:500
HA	11867423001, Sigma (Roche)	1:250
Mouse normal IgG	sc-2025, Santa Cruz	-
Rabbit normal IgG	sc-2027, Santa Cruz	-
